# Robotic-assisted revision total knee arthroplasty: a novel surgical technique

**DOI:** 10.1186/s42836-022-00160-5

**Published:** 2023-01-23

**Authors:** Hui-Ling Joanne Ngim, Dirk Van Bavel, Richard De Steiger, Andrew W. W. Tang

**Affiliations:** Epworth Healthcare, Melbourne, 3121 Victoria Australia

**Keywords:** Robotic surgery, Revision total knee arthroplasty, Surgical technique

## Abstract

**Background:**

Revision total knee arthroplasty is a challenging procedure. The robotic-assisted system has been shown to enhance the accuracy of preoperative planning and improve reproducibility in primary arthroplasty surgeries. The aim of this paper was to describe the surgical technique for robotic-assisted revision total knee arthroplasty and the potential benefits of this technique.

**Method:**

This single-centre retrospective study included a total of 19 patients recruited from April 1, 2021 to April 30, 2022. Inclusion criteria were patients who had Mako™ robotic-assisted revision total knee arthroplasty done within the study period with a more than 6 months follow-up. Statistical analysis was done using Microsoft Excel 16.0.

**Results:**

All 19 patients were followed up for 6 to 18 months. All patients in this study had uneventful recoveries without needing any re-revision surgery when reviewed to date.

**Conclusion:**

With the development of dedicated revision total knee software, robot-assisted revision TKA can be a promising technique that may improve surgical outcomes by increasing the accuracy of implant placement, and soft tissue protection and achieving a better well-balanced knee.

## Introduction

Revision total knee arthroplasty (TKA) is a challenging procedure, with a high complication and failure rate [1]. National joint replacement registries in Australia and Sweden have reported an increasing number of knee arthroplasty done annually throughout the last decade [2]. With the growing number of TKA done worldwide, a future increase is expected in revision TKA. From 2003 to 2017, the incidence of revision knee arthroplasty per 100,000 population increased from 11.7 to 19.5 in Australia, while over the same time span, in Sweden, the incidence rose from 6.6 to 9.4 [2]. Studies have shown that the reason for revision procedures is frequently caused by septic or aseptic loosening, instability, polyethylene wear, and osteolysis [3, 4]. Challenges in surgical techniques which may affect the outcome of revision total knee arthroplasty include careful preoperative planning, bone deficit management, soft tissue balancing and restoration of the joint line [5–7]. Robotic-assisted systems have been shown to enhance the accuracy of preoperative planning and improve reproducibility in primary arthroplasty surgeries [8]. The aim of this paper was to describe the surgical technique for robotic-assisted revision total knee arthroplasty from a TKA, unicompartmental knee arthroplasty (UKA) or second-stage revision knee arthroplasty.

## Materials and methods

This study was of retrospective design, with patients recruited from Epworth Richmond Hospital from April 1, 2021 to April 30, 2022. These patients were operated on by two senior surgeons experienced in revision arthroplasty surgeries and Mako™ robotic-assisted arthroplasty surgeries. The inclusion criteria were: patients previously having received TKA or UKA had Mako™ robotic-assisted revision TKA, or patients with cement spacer with well-controlled periprosthetic infection who received Mako™ robotic-assisted revision TKA. Patients whose follow-up time was less than 6 months were excluded. A total of 19 patients recruited fulfilled the inclusion criteria, including 12 females and 7 males, with their age ranging from 57 to 84 years old (mean 69.7 years old). All patients were revised using fully-cemented TKA implants from Triathlon revision knee system (Stryker Orthopaedics, Mahwah, NJ, USA) assisted by Mako™ robotic arm-assisted system. After revision surgery, patients were followed up at 6 weeks, at 3 months, at 6 months and 6 months after operation. AP and lateral radiographs of the knee were obtained during each follow-up to assess for any loosening or migration of implants. Patients' ambulatory status was assessed and monitored for any infection or any subsequent re-revision.

### Revision total knee arthroplasty to total knee arthroplasty

#### Preoperative imaging

**Fig. 1:**
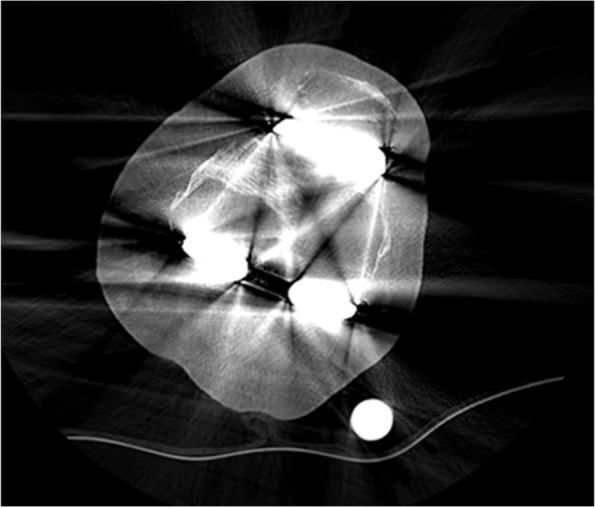
CT slice of a femoral component without metal artefact reduction software (MARS)

**Fig. 2:**
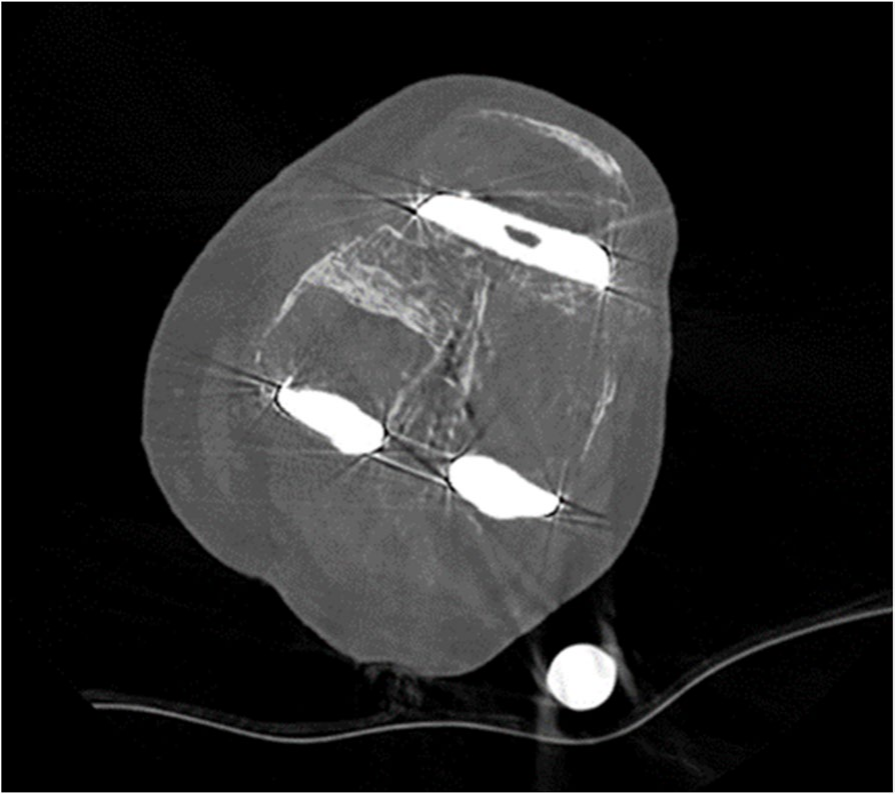
CT slice of the same level after MARS

Preoperative computer tomographic (CT) scan was performed using the Standard Makoplasty protocol. Patients were told to keep still to minimize the patients' motion artefact. Metal artefact reduction software (MARS) was used to reduce the metal artefact from existing implants to obtain a good-quality CT image. The CT scan was completed in a radiological center experienced in providing good metal artefact subtraction. This is vital to ensure the clear visualization of the implant, especially the femoral component (Figs. 1 and 2). The major concern regarding a revision total knee arthroplasty is the accuracy and reliability of bony registration, with CT imaging compromised by metal artefact from the *in situ* prosthetic components. The CT scan images were manually segmented by the Mako Product Specialist (MPS) before being uploaded into the robotic system.

#### Preoperative planning

**Fig. 3:**
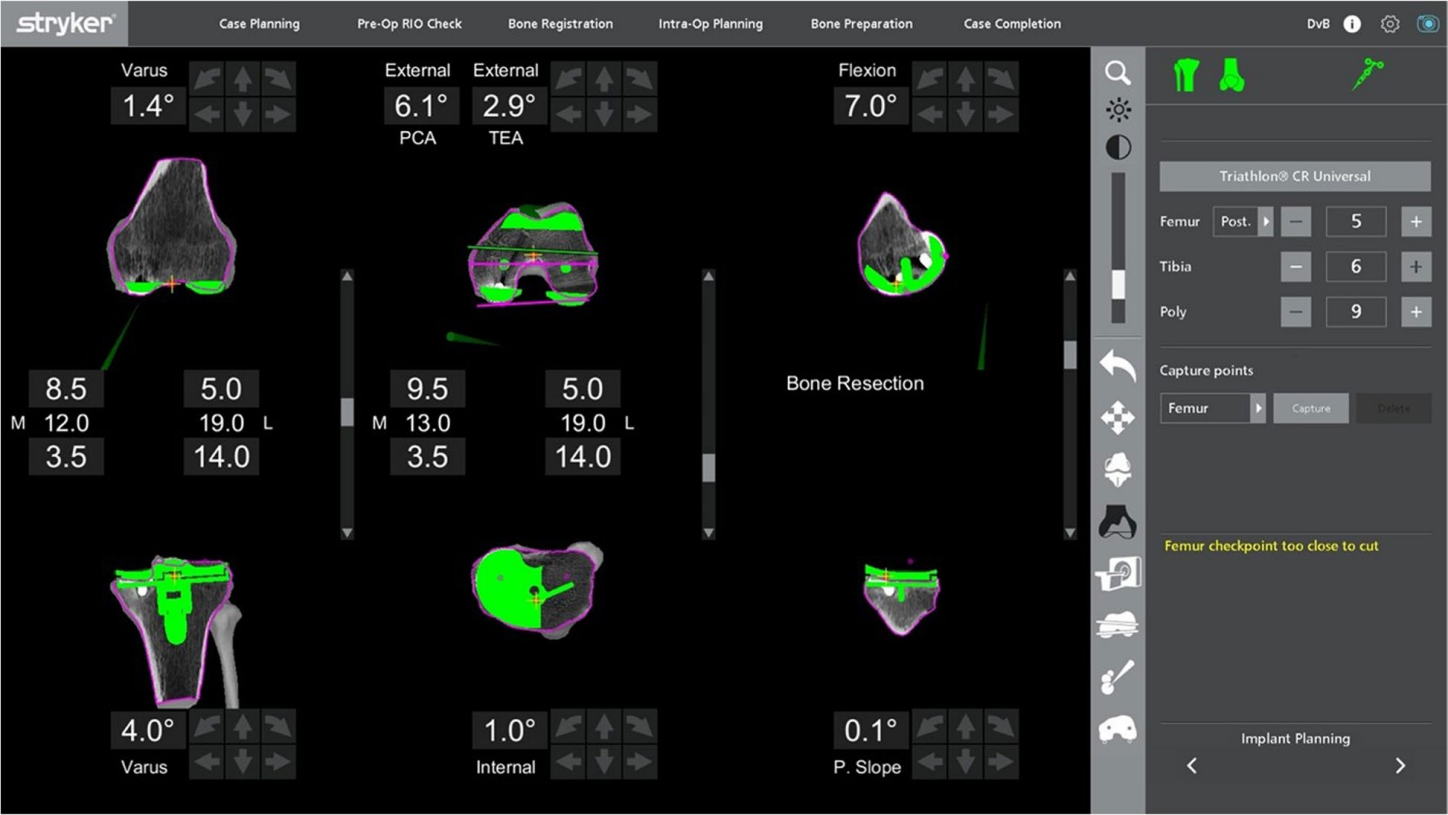
Scaled image of the preoperative plan for visualization of stem placement

A preliminary preoperative planning was performed on the Mako robotic system. The main aim of this plan was to ensure that the joint line was maintained at its original level in relation to the femoral medial epicondyle and the fibular head. This was achieved by planning for a minimal bony resection at both distal femoral condyles to limit distal femoral bone loss. This planning page was also useful to determining the implant size and the most ideal placement of the new implant, by enabling visualization of the position of the implant stem in the femoral and tibial canal. By scaling the image (Fig. 3) and measuring it with a ruler, the desired length of the stem was determined. However, in many cases, there was a need for intraoperative adjustment of the plan after removal of the existing implant based on bone loss.

### Intraoperative processes

#### Placement of the array

**Fig. 4:**
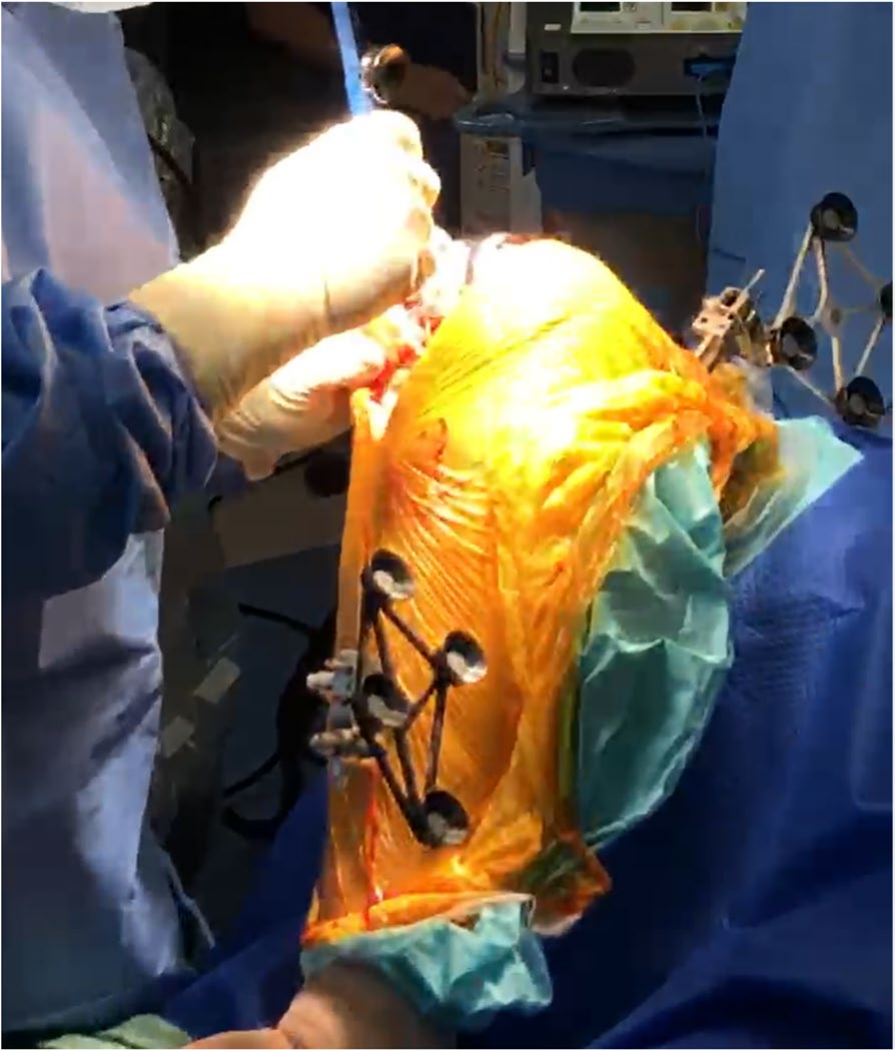
Placement of femoral and tibial pin with arrays placed away from the knee incision

The femoral and tibial array pins were placed through separate stab incisions away from the knee wound (Fig. 4). This ensured that there was an adequate length of the bone to accommodate stems in both the femur and tibia.

#### Bony landmark registration

**Fig. 5:**
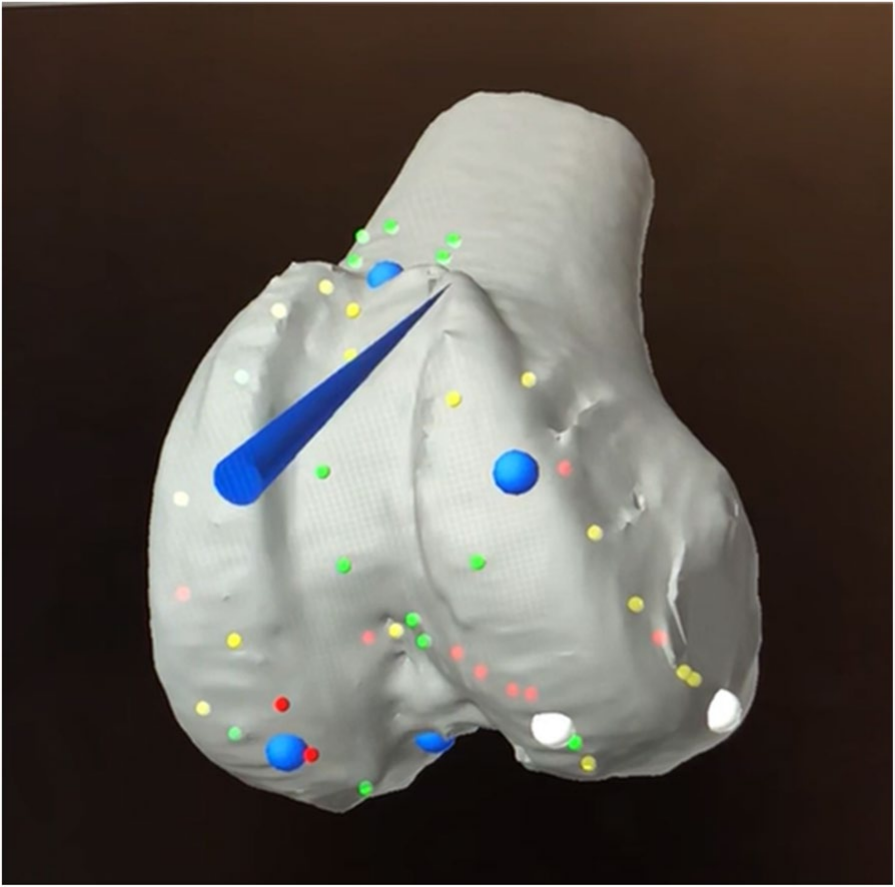
Femoral bony landmark registration with a total knee femoral component *in situ*

Registration of the femoral bony landmarks was completed without the removal of the existing implant. The femoral registration was performed on the bone at the periphery of the femoral component mainly at both the lateral and medial edges of the femoral component, the notch and anterior femur being proximal to the anterior flange (Fig. 5). A proposed standard registration pattern may not be possible because some points may be affected by the metal artifacts or obscured by the implant. Therefore, other additional points on bony surfaces can be obtained.

**Fig. 6:**
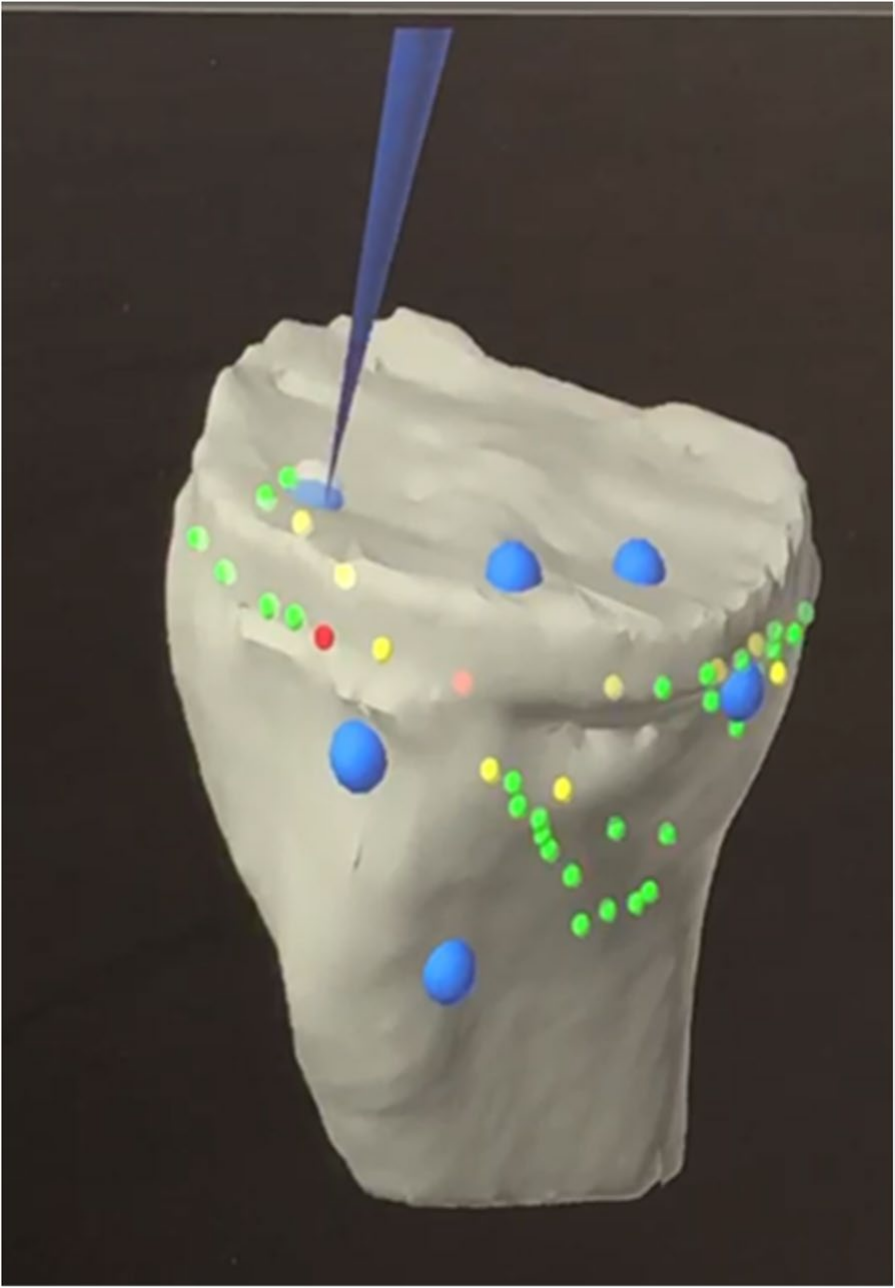
Tibial bony landmark registration with a total knee tibial tray *in situ*

The polyethene insert was then removed using a standard method. Registration of the tibial bony landmark was performed with the existing tibial tray *in situ*. As with the femur, the points for registration of the tibia were at the periphery of the tibial tray, both at the medial and lateral proximal tibia, as well as the anterior tibial metaphysis around the tibial tuberosity. Registration and verification on the top of the tibial tray were used in some of the cases (Fig. 6). The tibial registration and verification were noted to be easier and could achieve higher accuracy when compared to the femoral landmark registration.

#### Ligament balancing and removal of metal components

A trial insert was reinserted back for ligament balancing prior to the removal of the metal components. Removal of the femoral component was done by using the standard method to minimize bone loss typically associated with a fine saw and osteotomes.

Thee tibial tray was removed with the Mako saw, with the tibial cut level just away from the tibial tray and saw anterior to and around the tibial tray keel. And then the remaining attached surfaces were cut.

#### Intraoperative adjustment of the plan and execution of bony cuts

**Fig. 7:**
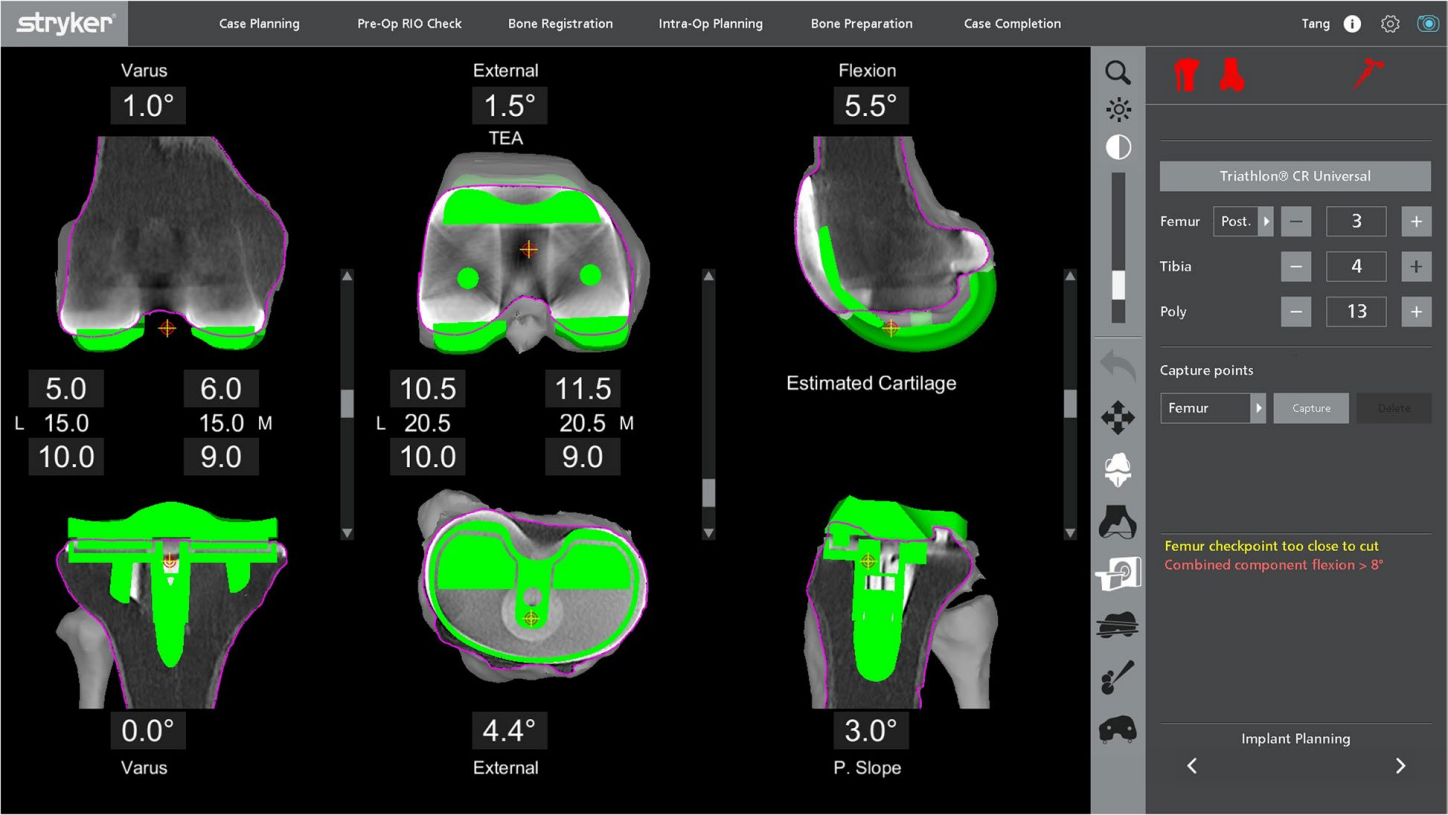
Preoperative planning for a patient with an over-sized femoral component with patella baja

After removal of the metal components, bone loss was assessed visually before proceeding to the bony cuts. The sequence of the bone cuts was the same as that in a primary knee case, where the right-angle saw was used first for the distal femoral cut, then the posterior chamfer cut, followed by the sagittal sawing for the remaining cuts. If the bone loss was minimal and augments were not necessary, refresher cuts were performed to the femur and tibia. This was achieved by making adjustments to the preoperative planning page to enable a sliver of bone to be cut at the distal femur. The posterior femoral chamfer cut will be adjusted accordingly. In a patient with an over-sized femoral component and distal femoral bone defect with patella baja, distal femoral augments were needed to distalize the joint line (Fig. 7). In the cases where bone loss was affecting one condyle, the plan was adjusted to proximalize the component by a 5-mm increment until the desired cut level is obtained. The level was returned to the initial level before proceeding to the next cut. This allowed for the use of a 5-mm augment.

**Fig. 8:**
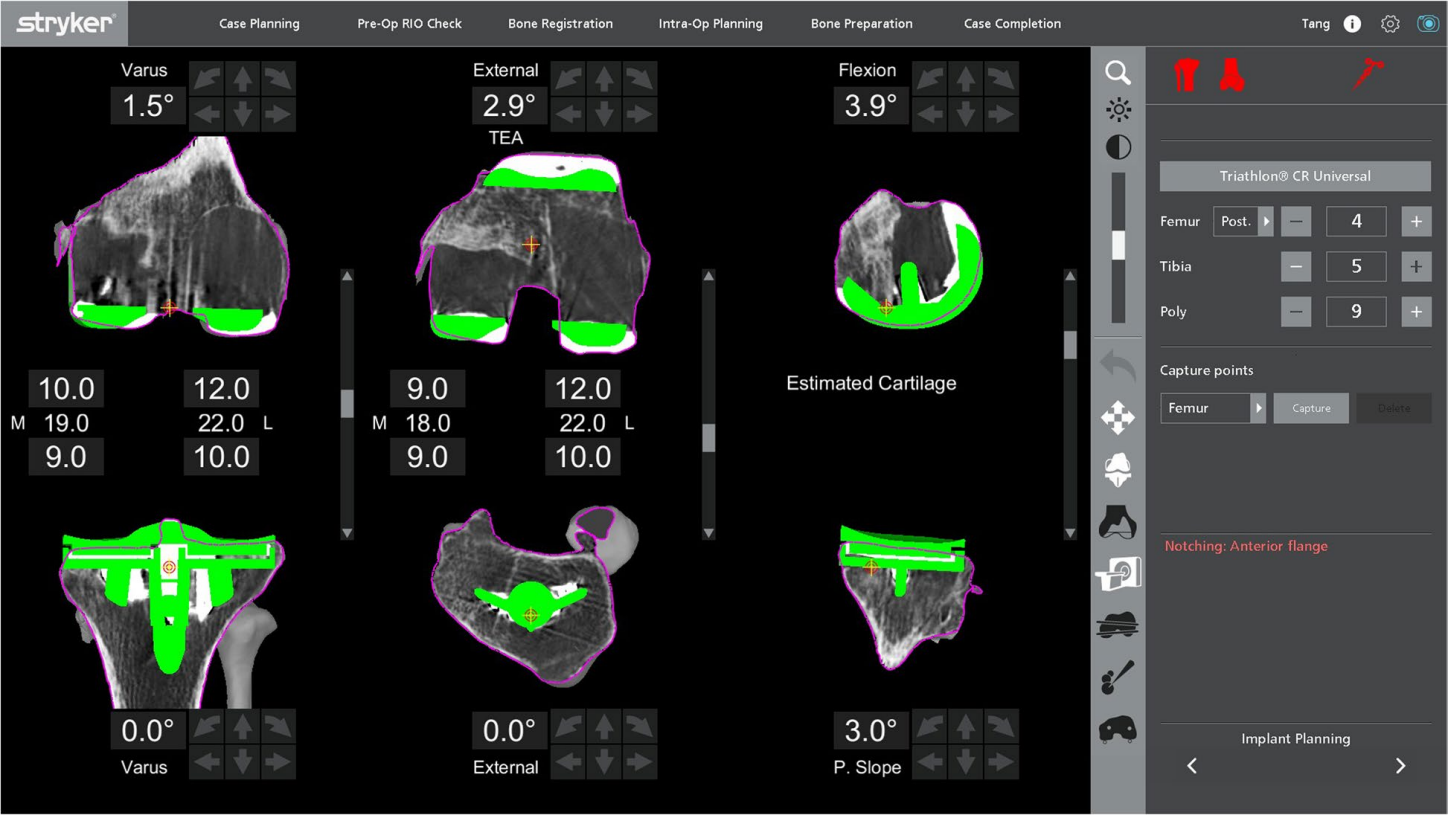
Preoperative planning of a patient with massive bone cyst involving both femoral condyles

After changing to the sagittal saw, the anterior femur was cut first to prevent any notching from occurring, before proceeding to the anterior chamfer cut and, lastly, the posterior condyle cutting was done. In the presence of bony defect in the posterior condyles (Fig. 8), intraoperative adjustment of the plan was to accommodate augments at the posterior condyle. This was achieved by anteriorizing the cut in 5-mm increments until the desired cut level was obtained. With every alteration to the plan, the green probe was used to recheck the checkpoint & saw blade prior to performing the bone cut. The femoral box cut and stem preparation were completed using conventional instruments. The tibia refresher cut was set by adjusting the planning page to achieve the optimal cut level.

#### Ligament balancing and implantation of final implants

After completing all the bone cuts, trial components were inserted. Ligament balancing was possible using the ligament balancing page in the robotic system when real-time feedback of the gaps and range of movement of the knee was provided. Appropriate releases were performed to balance the knee. This was followed by standard steps of a total knee arthroplasty like cutting the tibial keel, femoral lug holes, resurfacing the patella and final component implantation. Lastly, the checkpoints and array pins were removed.

### Revision unicompartmental knee arthroplasty to total knee arthroplasty

#### Preoperative imaging

Similar to cases of revision from a total knee arthroplasty, Metal Artefact Reduction Software (MARS) for the Mako CT scan was used to obtain a good visualization of the existing implant, which could bring about a better segmentation. This was found to be particularly important for the femoral component because artefacts tend to be more scattered around the femoral component than the tibial component.

#### Preoperative planning

Component planning depended on the degree of anticipated bone loss and the surgeons' alignment philosophy. Revision type implants including stems and augments were prepared and readily available. The most commonly used extras are a medial tibial augment, usually a 5-mm augment and a short tibial stem.

With careful removal of the existing unicompartmental total knee femoral component, a primary femoral component can be used in most cases of revision from a UKA to TKA. This is because the femoral component from a unicompartmental knee implant is often thinner than a primary knee replacement femoral component. In some cases, the femoral component was deliberately flexed to better compensate for the bone loss on the posterior femoral condyle. In order to help minimize this bone defect and to avoid the need for an augment, the femoral component might also be anteriorized by 1 or 2 mm.

**Fig. 9:**
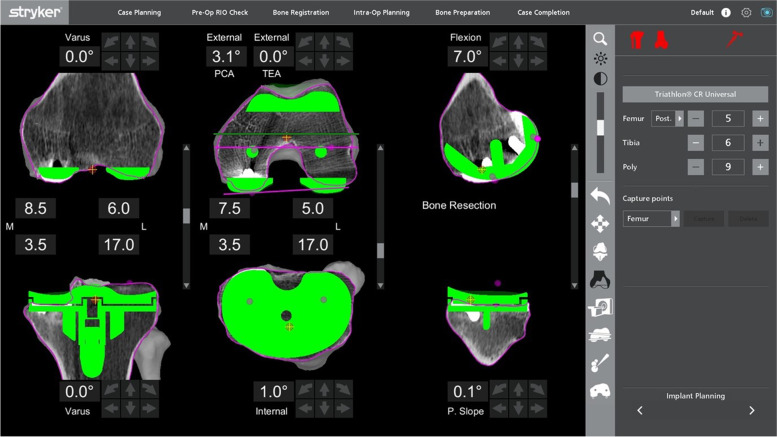
Preoperative planning of a patient with existing UKA revised to a TKA (a mechanically-aligned knee plan)

For the medial tibial cut planning, the tibial component was raised and lowered to find the ideal level. The slope of the tibial cut was altered to match what was required by viewing the slope of the current implant on the sagittal planning screen. Figure 9 shows the planning page of a patient who underwent a revision UKA to TKA, planned for a neutral mechanically-aligned tibial cut. In order to achieve a cut below the tibial component, a cut of 3.5 mm below the upper surface of the component was needed, and this corresponded to a 17-mm lateral cut. A medial 5-mm augment could be planned to minimize bone loss, correlated to a 12-mm lateral cut, which then would require a 12- or 13-mm polyethylene insert.

**Fig. 10:**
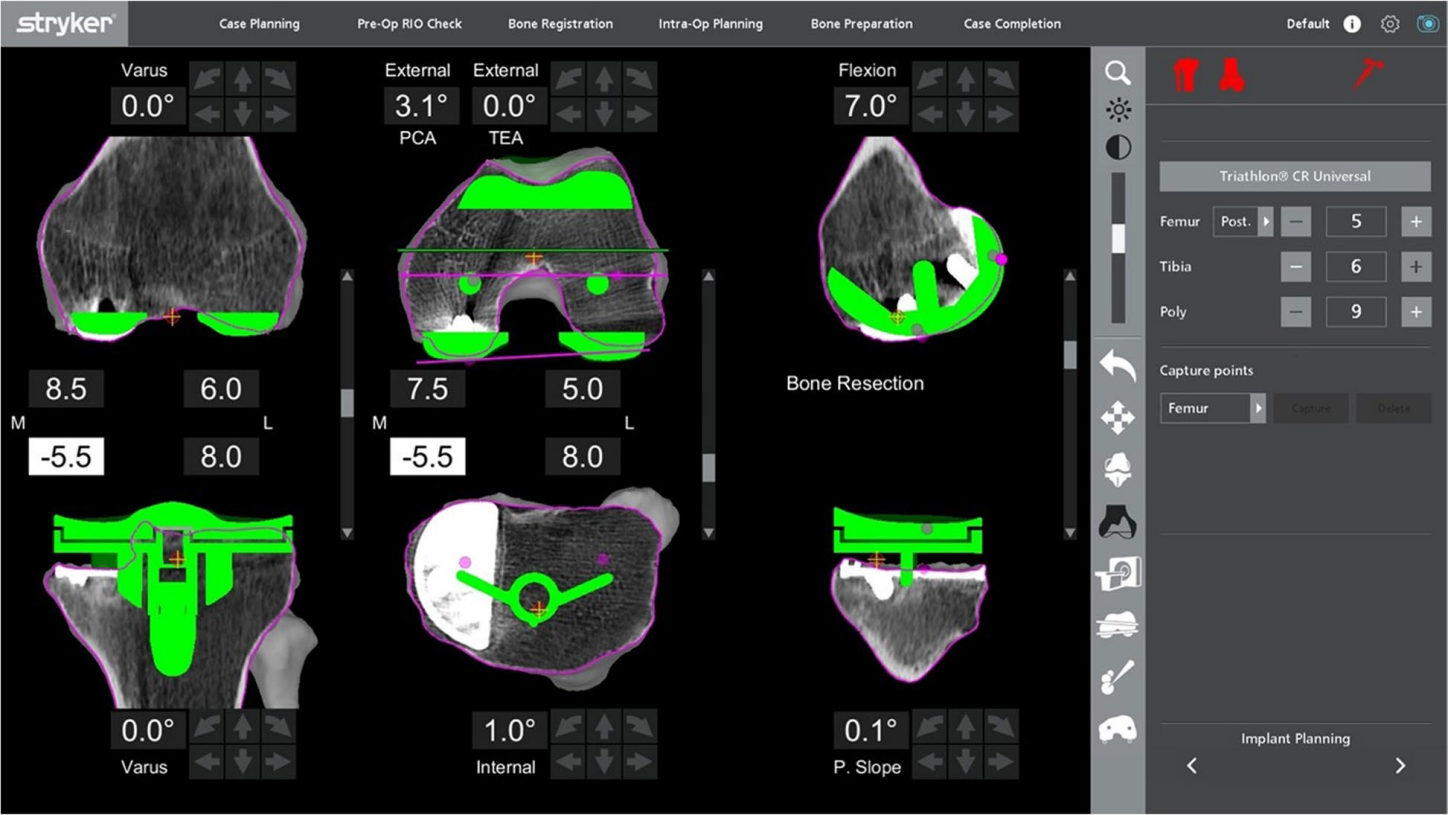
Alternative mechanically-aligned knee plan for the patient in Fig. 9

Alternatively, a slightly lower medial cut could be planned to get to fresh bone under the component. This would be the equivalent of 18 mm off the lateral side. Subsequently, a 10-mm medial augment would be needed (by raising the plan by 10 mm, Fig. 10), correlating to a normal lateral tibia cut requiring a standard 9- or 10-mm insert.

**Fig. 11:**
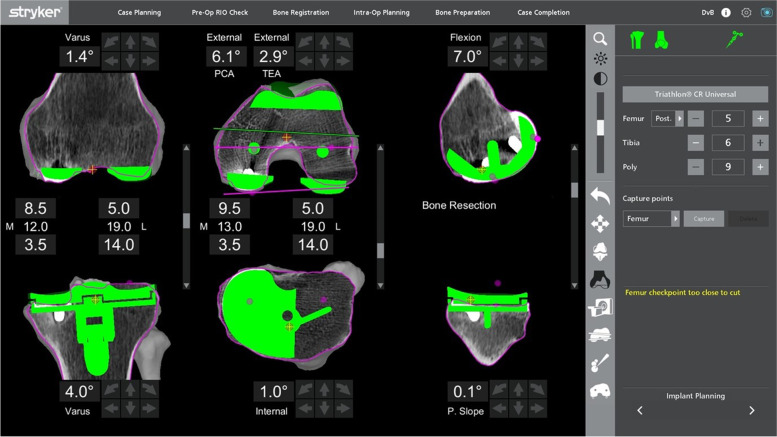
The non-mechanically-aligned knee plan for the same patient in Fig. 9

With this robotic system, a non-mechanically-aligned knee could also be planned (Fig. 11). Most typically, this would involve cutting the tibia with a few degrees of varus. Typically, the knee was planned with 20 mm gaps medially and laterally in flexion and extension for a 9-mm insert. In this case, the cut was dropped by 2 mm as there was inadequate bone cut from the medial tibia, which allowed for a planned 11-mm polyethylene insert. The lateral gap was decreased by not cutting the distal femoral cut in valgus and slightly externally rotating the femoral component.

#### Placement of the array

**Fig. 12:**
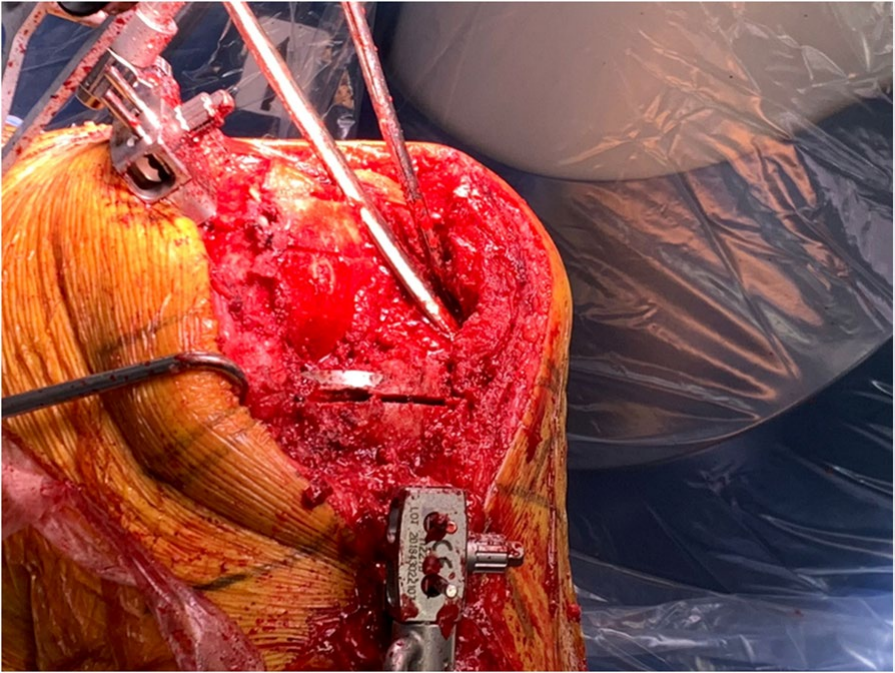
Placement of array pins in knee incision. Existing tibial tray was removed by using the Mako saw to cut distal to it

The femoral and tibial array pins could be placed in the same incision (Fig. 12) as the knee if no stems were planned. Alternatively, they could be placed in separate stab incisions at the diaphysis if stem insertions were anticipated.

#### Registration of Bony Landmarks

**Fig. 13:**
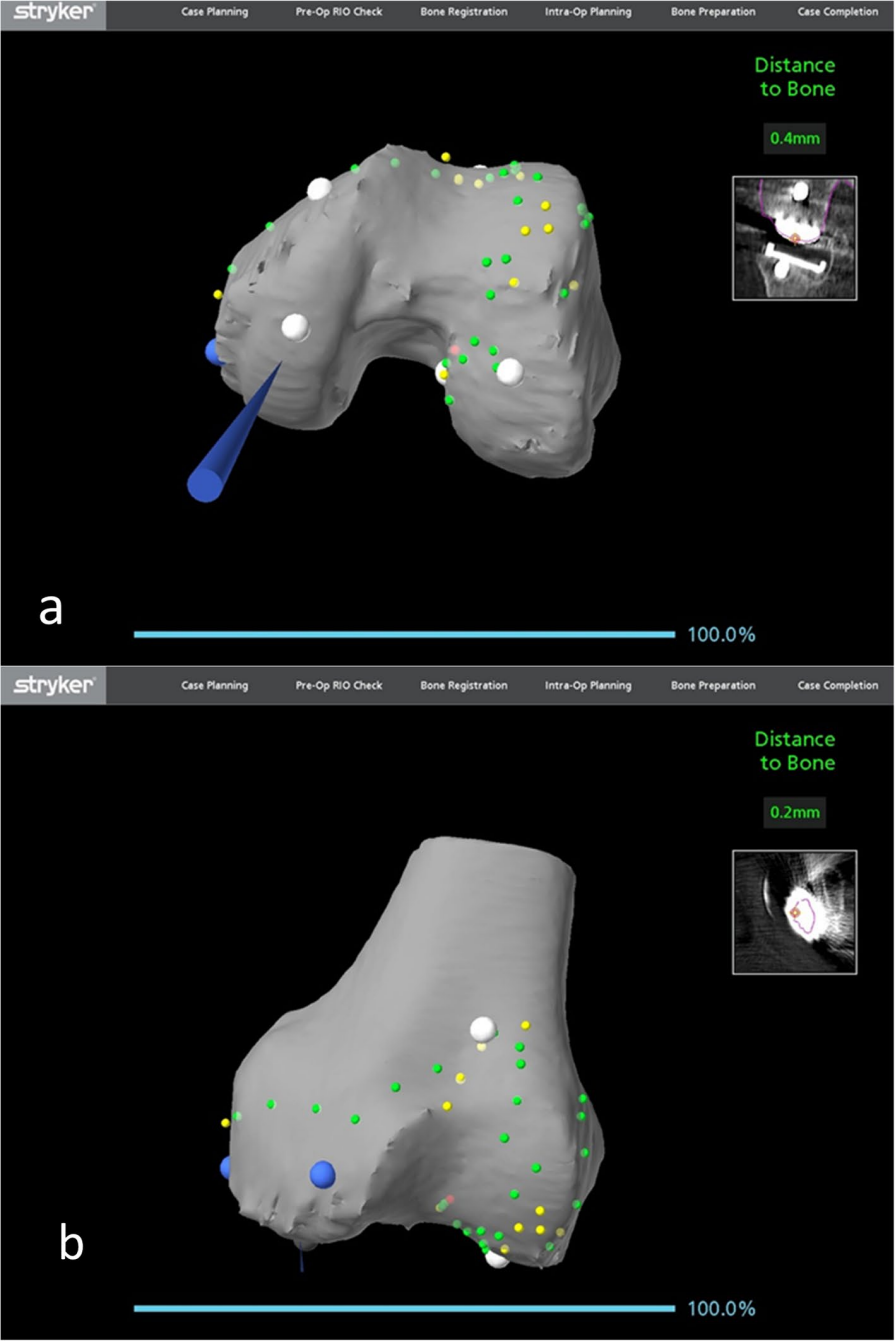
Femoral bony landmark registration with a medial unicompartmental knee femoral component *in situ*

Registration of bony landmarks for the cases of conversion of a medial or lateral unicompartmental knee arthroplasty to a total knee arthroplasty was similar as revision from a total knee arthroplasty to a total knee arthroplasty. Bone registration mainly involved plotting points on the native femoral condyle articular surface, the trochlea and anterior femur and along the medial condyle away from the articular surface with good overall accuracy (Fig. 13a, b).

**Fig. 14:**
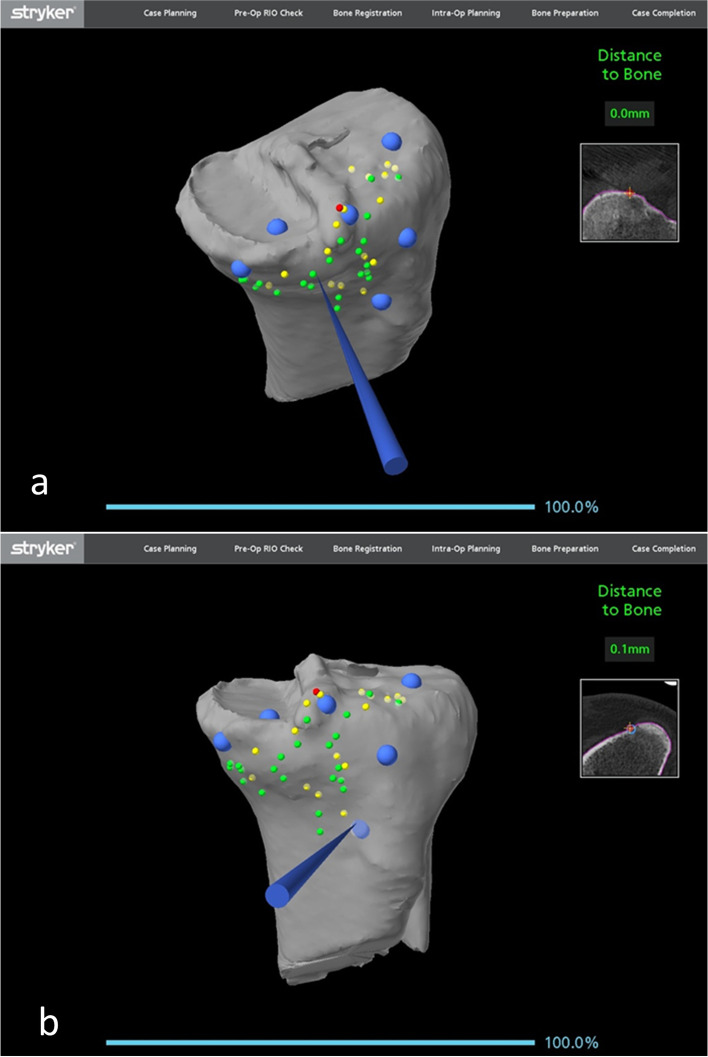
Tibial bony landmark registration with a medial unicompartmental knee tibial tray *in situ*

While tibial registration could be performed on the superior surface of the component once the insert was removed, this was found to be unnecessary. By working on bony landmarks on the lateral tibial plateau, anterior to tibia and medially away from the prosthesis, satisfactory accuracy of registration was achieved (Fig. 14a, b).

#### Removal of metal components and execution of bony cuts

The femoral component was routinely removed with a microsaw or fine osteotomes, ensuring minimal bone loss to allow for the use of a standard primary prosthesis. On the tibial side, the component could be removed with routine instruments as well but often was removed with cuts performed by using a Mako saw. The Mako saw was used to cut anterior and lateral to the keel (Fig. 12) before completing the lateral cuts, and, if possible, the saw should go posteriorly just lateral to the keel. It is more difficult to cut adjacent to the medial side of the peg and keels due to the restrictions from the haptic boundary. This may be improved by expanding the boundaries and using the narrow Mako saw blade. To use a narrow saw blade, intraoperative adjustment to the planning page was necessary by down-sizing the tibial component to size 1 or 2, which triggers the system to change the saw blade. With the knee placed in a hyper-flexed position, the Mako narrow saw blade was found to be helpful for seeing around the keel. However, the cuts might need to be completed using a microsaw to access the posteromedial corner of the tibial plateau in most cases. Care was taken to ensure that the saw was not damaged on the implant when the cut was performed.

When performing a step cut for an augment, the robot was set for the lesser lateral cut first. The plan was then dropped for the augment (typically by 5 mm which is the size of the augment) and the medial side was cut, with care exercised not to undermine the lateral bone. The bone cut can be observed on the screen and care was taken to only cut, at the deeper level, the amount of bone required, starting from medial to lateral, to fit the required augment.

After finishing the bone cuts, re-assessment of the soft tissue balance was completed with the trial implants using the ligament balancing page, thereby giving real-time information of the gaps and range of movement of the knee. Standard steps of a total knee arthroplasty were then performed by cutting the keel and femoral lug holes, resurfacing the patella and implanting the final implants.

### Second stage revision from a cement spacer to a total knee arthroplasty

#### Preoperative imaging

A preoperative CT using standard Makoplasty protocol was required for such cases. In view of the absence of metallic implants for such cases, no metal artefact subtraction was needed.

#### Preoperative planning

**Fig. 15:**
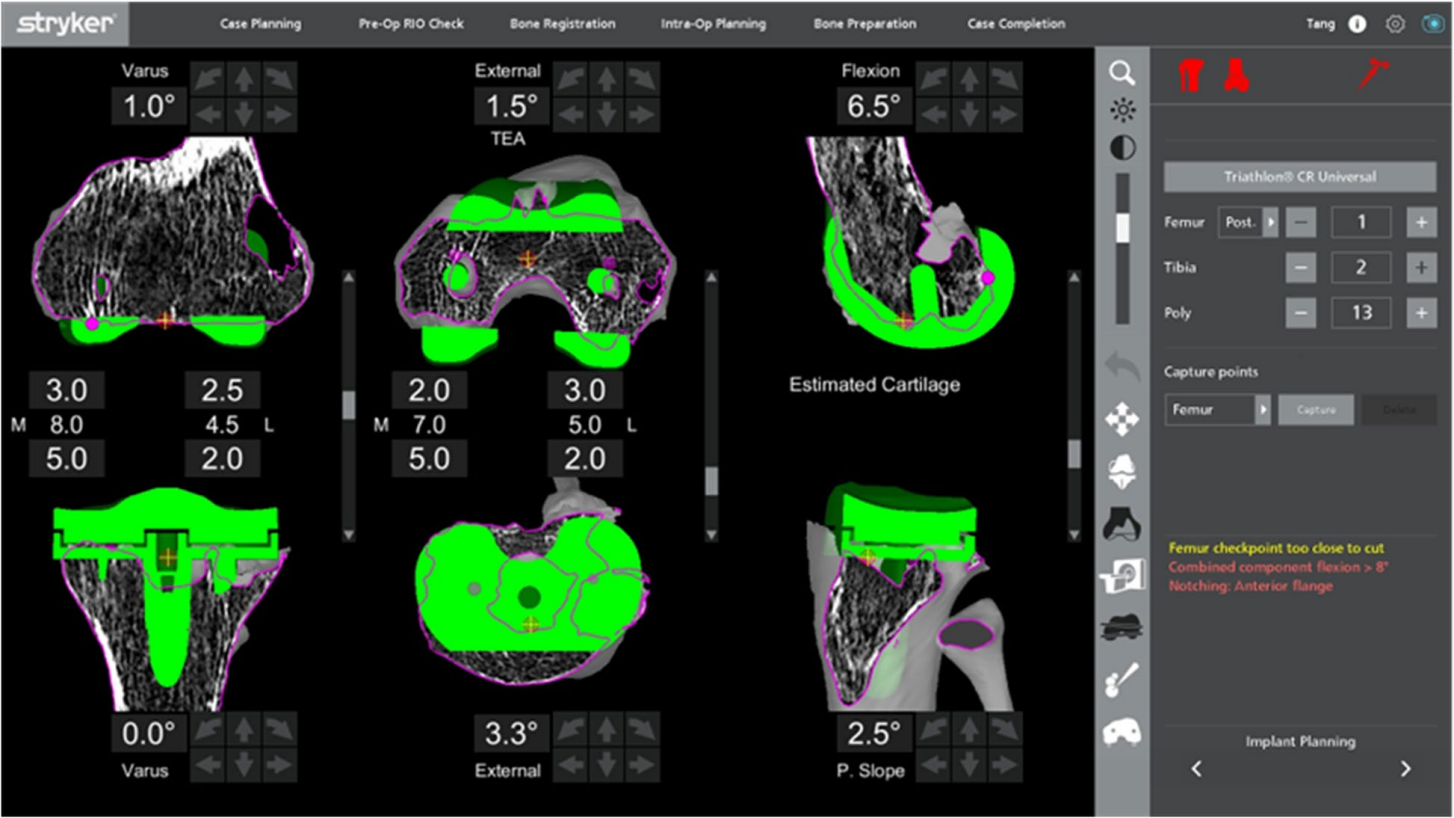
Preoperative planning of a patient with antibiotic cement spacer for periprosthetic joint infection

The preoperative planning page was used to identify the native joint line as many bony landmarks might be difficult to identify in such cases due to bone loss and scarring. The epicondyles, in particular, are often easier to identify on the CT scan than intraoperatively. Femoral augments can be planned to build up for bone loss accordingly to prevent elevation of the joint line. This page was also helpful to determining the placement of the implant so that the stem was placed in the center of the canal, thus avoiding impingement on the cortex (Fig. 15). In cases where tibial bone loss is present, tibia augments can be used. Alternatively, a thicker polyethene can be used in cases where equal bone loss occurs at both medial and lateral parts of the proximal tibia.

#### Placement of array

The pins for the arrays were placed in separate stab incision at the femoral and tibial diaphysis due to the high possibility of using stems.

#### Bony landmark registration

Bony registration for both the femur and tibia was obtained on the bone away from the cement spacer if it was not cemented onto the bone. However, if the cement spacer is cemented onto the bone, it is more ideal to do the bony landmark registration prior to removal of the spacer because further bone loss might occur during the removal, and this may affect the accuracy of the registration and verification of the bony landmark.

#### Bony cuts

Bony cutting for the implants and augments can be adjusted on the planning page to achieve refresher cuts to minimize further bone loss. The sequence of the cuts was the same as is used in a primary total knee arthroplasty with the right angle saw, followed by the sagittal saw. Augment preparations were completed at the distal femur by adjustment of 5 mm increment to accommodate to the bone loss. Similarly, in the posterior femoral condyle, bone loss needing augments required augment preparation, with intraoperative adjustment of the planning page by 5 mm increment to fit the appropriate augment. Chamfer cuts were performed at the corresponding levels.

#### Trial implant placement and ligament balancing

Trial implants were inserted for ligament balancing. Real-time feedback on the gaps throughout the range of movement shown in the robotic system was helpful to achieve a well-balanced knee. Objective measurements of gaps and alignment provided were found to be more helpful than solely relying on the traditional gap-balancing technique.

**Fig. 16:**
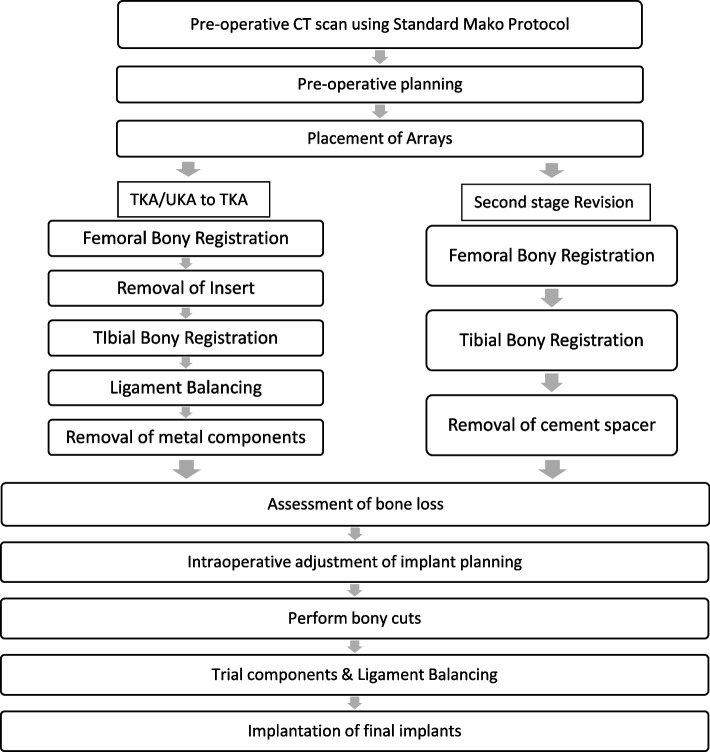
Steps of surgical techniques for robotic-assisted TKA

During the placement of the trial implant specific to the tibial tray, the robotic system was used to place it in the correct rotational alignment. This can be achieved by using the green probe to show the midline of the tibial tray to help its alignment on the tibia. The green probe can also be used to check the medial/lateral and anterior-posterior positioning of the trial component. This was found to be useful in the cases where boss loss and scarring might distort the native anatomy and important bony landmarks. The total knee arthroplasty was completed after keel and femoral lug preparation, patellar resurfacing and implantiation of the final components. The steps of the surgical technique are summarized in Fig. 16.

## Results

Of the total 19 patients, 12 cases were revised from a primary TKA (11 cases of aseptic loosening, one unbalanced knee with restricted range of movement). Four cases were revised from UKA (two cases due to polyethylene wear and two due to aseptic loosening), three cases were periprosthetic joint infection of a previous TKA whereby the first-stage revision with cement spacers was done. The patients were followed up for 6 months to 18 months (mean, 10.4 months). All 19 patients were able to achieve independent ambulation in their community. The mean range of motion for the knee during the final review was 1.5 degrees flexion (range, 0 to 5 degrees flexion) in full extension and 114 degrees flexion in full flexion (range, 100 to 130 degrees flexion). They also experienced uneventful recoveries without any infections nor needing any subsequent re-revision surgeries to the latest review.

**Fig. 17:**
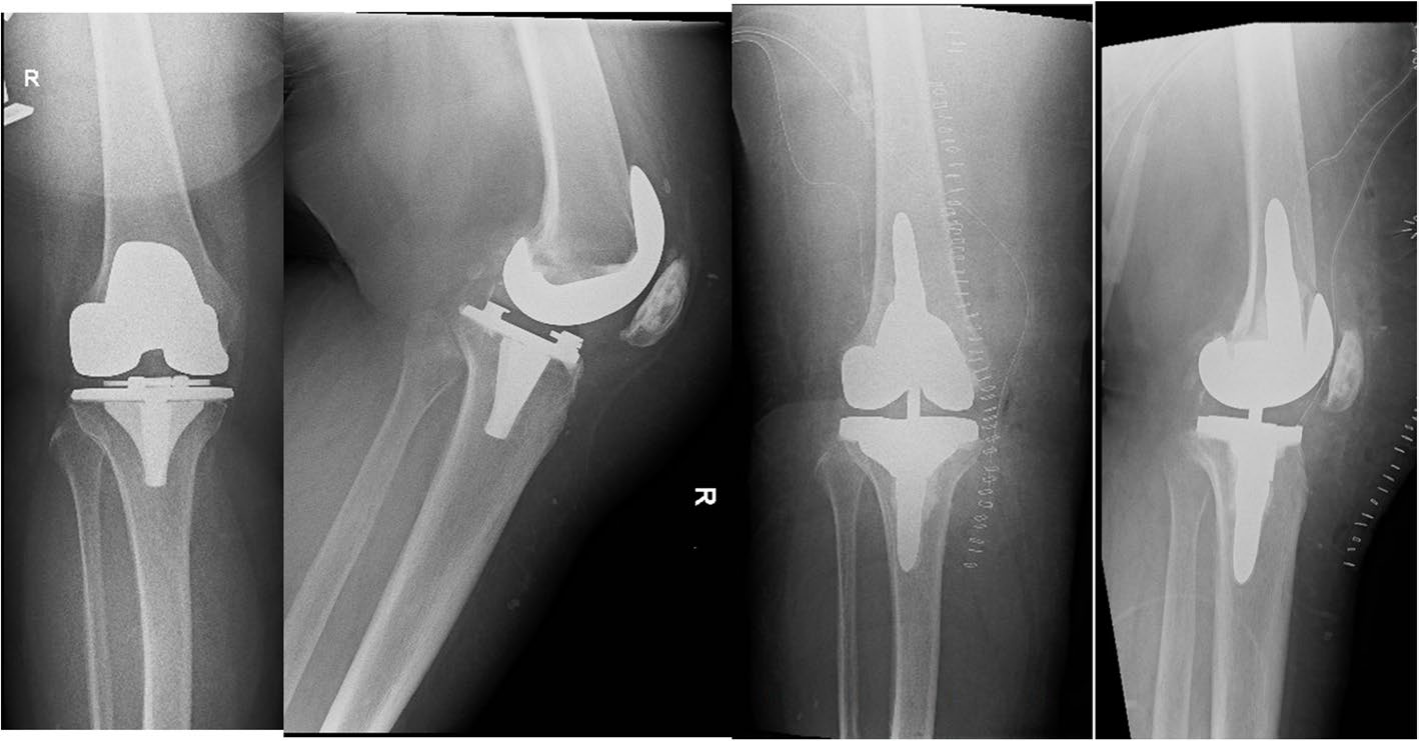
Pre- and postoperative radiographs of patient for preoperative planning in Fig. 7

**Fig. 18:**
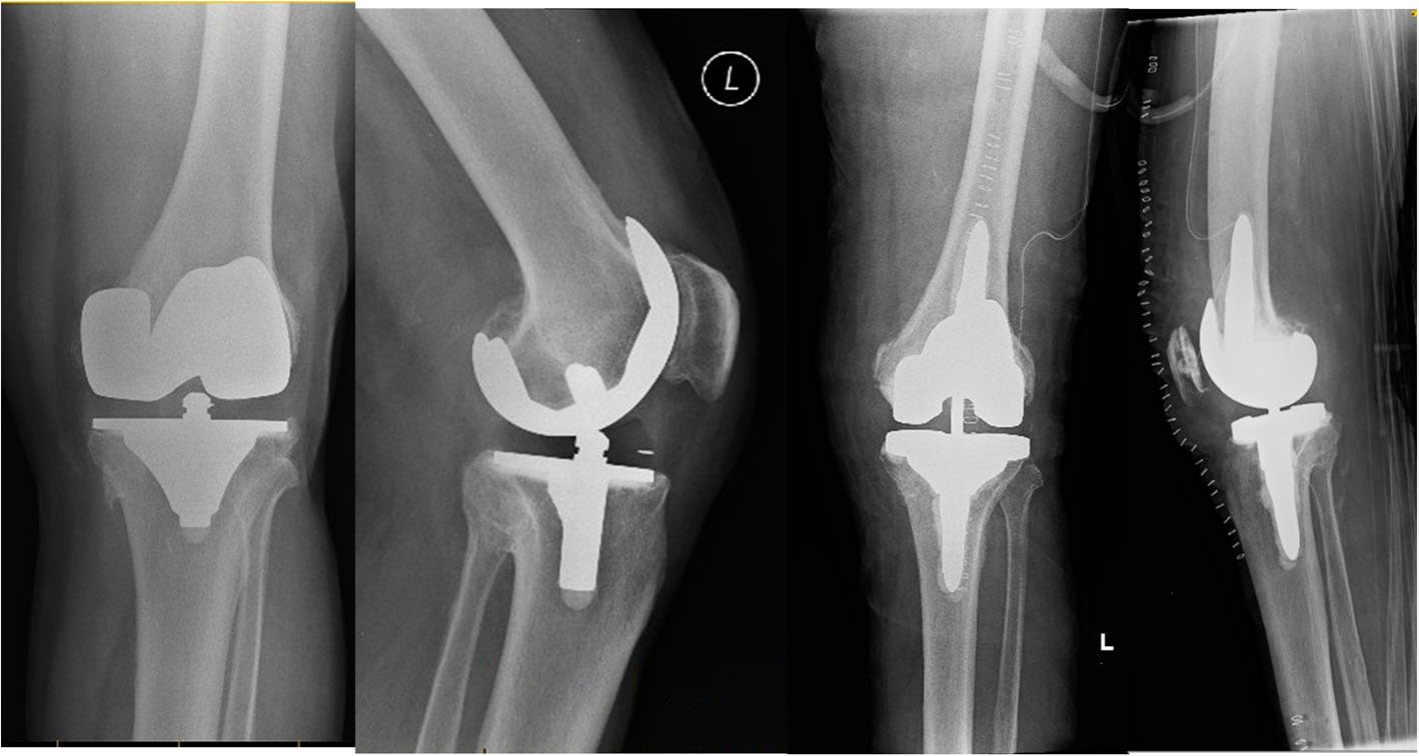
Pre- and postoperative radiographs of patient for preoperative planning in Fig. 8

**Fig. 19:**
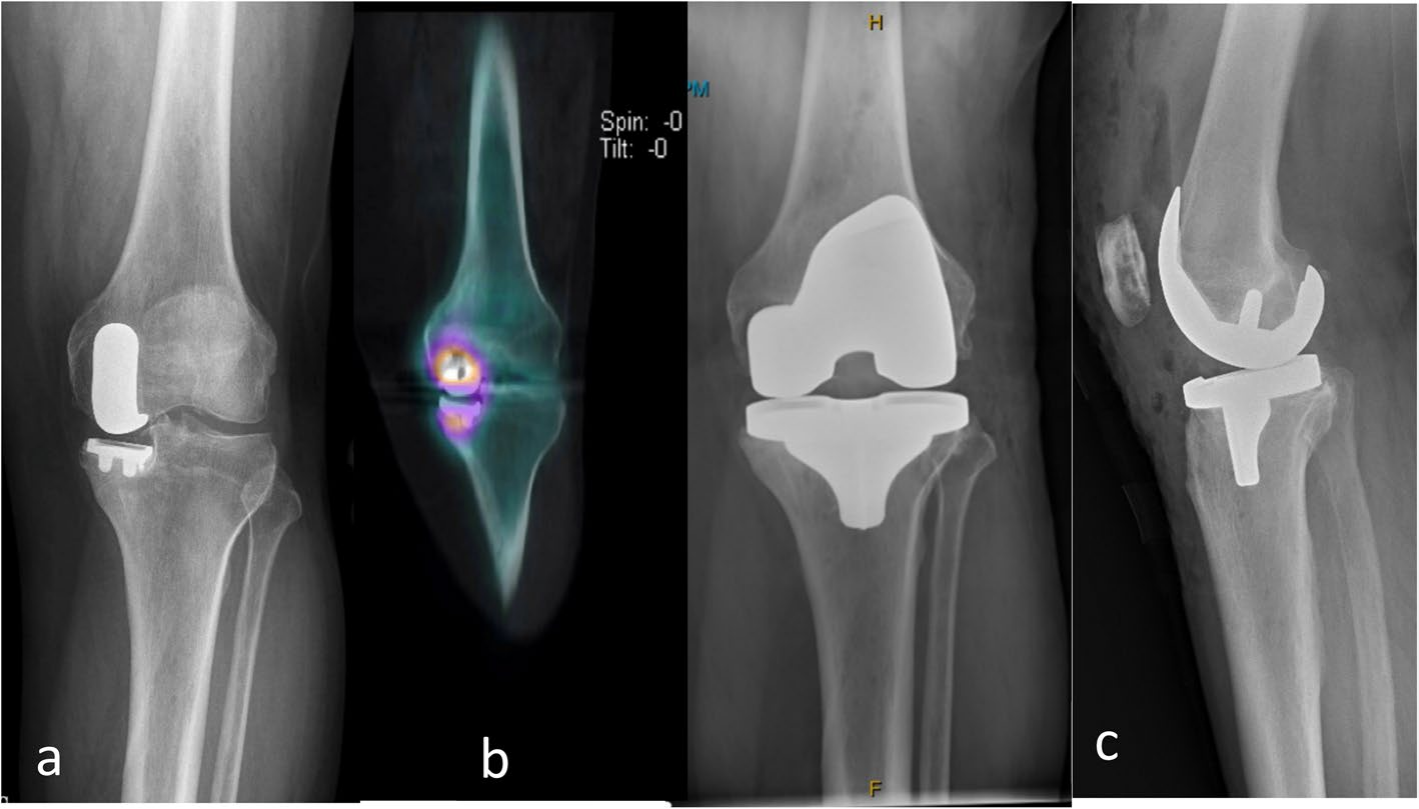
**a** Preoperative knee radiograph of the patient with the preoperative plan from Fig. 11; **b** Spect CT of the knee of the same patient showing loosening of the UKA; **c** Postoperative knee radiograph of the same patient

**Fig. 20:**
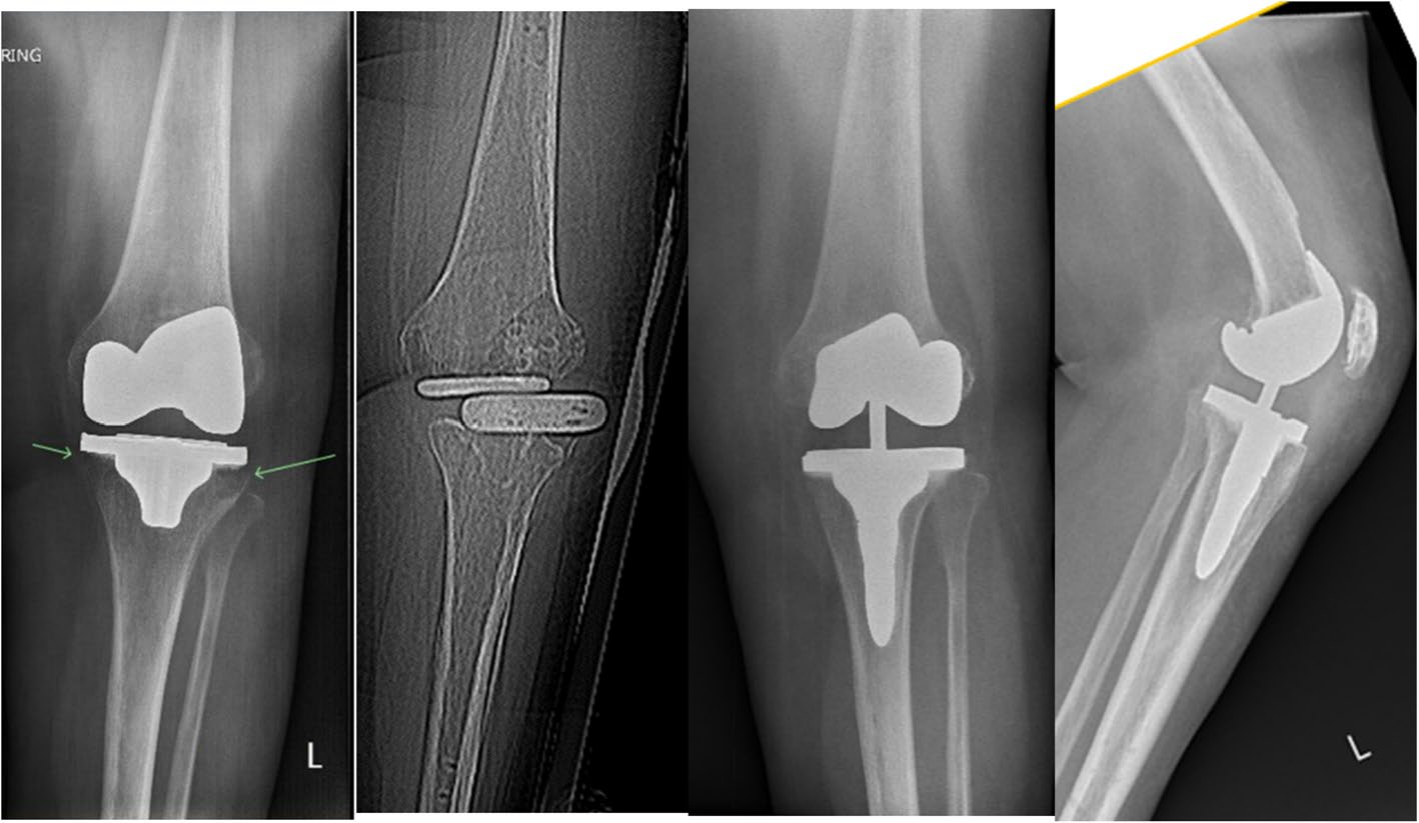
Pre- and postoperative radiographs of the patient for the preoperative planning in Fig. 15

Both patients whose planning page are shown in Figs. 7 and 8, had aseptic loosening of their primary TKA as the reason of the revision. The pre- and postoperative radiographs of the knees of these two patients are shown in Figs. 17 and 18 respectively. For the patient mentioned in the revision from a UKA to a TKA (planning page shown in Fig. 11), the reason of revision was also aseptic loosening. The pre- and postoperative radiographs of the patient from Fig. 11 are shown in Fig. 19. The pre- and postoperative knee radiographs of the patient with antibiotic cement spacer treated for periprosthetic joint infection who had undergone a second stage revision TKA are shown in Fig. 20.

## Discussion

Robotic-assisted primary knee arthroplasty has been shown to increase accuracy and precision in bone cuts and component position to plan compared with conventional manual-jig technique [9]. There are also studies which demonstrated that robotic-assisted knee arthroplasty had an increased accuracy of alignment with regards to reconstituting a neutral mechanical axis and minimizing a number of outliers on the coronal plane [10]. Therefore, in revision knee arthroplasty surgery, the placement of the component should be accurate and precise. This can be achieved with the guidance of robotic technology.

Restoration of the position of the joint line to the same level as the native joint line is one of the important principles of primary and revision total knee arthroplasty [11]. Due to the lack of anatomical landmarks in revision arthroplasty cases, the joint line is frequently malpositioned [12]. Commonly used surgical techniques, especially the ones based on the balancing of the flexion and extension gaps, frequently resulted in elevation of joint line [13]. An elevation in the joint line, compared to the contralateral healthy knee, has been shown to result in poorer functional outcomes [14]. Therefore, it is of utmost importance to restore the joint line in revision arthroplasty to obtain a good functional outcome. Agrawal *et al*. reported that robotic knee arthroplasty significantly increased accuracy and achieved an almost anatomical position of the joint line in primary knee arthroplasty when compared to the conventional method [15]. Using the image-based robotic system, the joint line can be determined through the preoperative planning page using the femoral adductor tubercle instead of using the commonly used balancing technique based on the tibia, which may help in the restoration of the level of the native joint line.

Other benefits of using robotics in revision arthroplasty include soft tissue protection. A review by Sultan concluded that robotic-arm-assisted TKAs were comparable or superior to manual TKA in soft-tissue protection [16]. A cadaveric study by Hampp reported that damage to the posterior cruciate ligaments was significantly less in primary robotic knee arthroplasty when compared to the conventional instrumented method [17]. It is postulated that the enhanced preoperative planning with the robotic software, the real-time intraoperative feedback, and the haptically-bounded saw blade, may all help protect the surrounding soft tissues and ligaments [17]. In addition, Clark reported less opioid use, reduced pain, and shorter length of stay postoperatively in patients who underwent primary robotic knee arthroplasty compared with those who received computer-navigated knee arthroplasty, thus resulting in improved short-term patient outcomes with robotics, which can be ascribed to reduction in iatrogenic soft-tissue damage and preservation of vulnerable structures in the knee [18]. This benefit can only be attained by using the haptic feedback available in robotic technology and not other methods.

Using the robotic system can also help achieve a balanced knee. Traditional methods of component alignment and ligament balancing are either performed without objective measurement or are subject to measurement errors [18]. With the robotic system, gaps can be planned during the preoperative planning. Sensor feedback in the system can provide real-time information thus helping surgeons achieve a well-balanced knee throughout the range of movement. Recent studies of robotic-assisted primary TKA showed that surgeons were able to achieve quantitatively balanced knee by using the robotic platform with real-time feedback from intraoperative load sensors [19, 20]. A study by Held *et al*. reported that robotic-assisted TKA resulted in improved intraoperative compartment balancing when compared with conventional TKA [21]. This objective feedback system from the robotic-assisted technology can help attain a more balanced knee in revision knee arthroplasty cases, especially when surgeons are less experienced.

We are aware that the main limitations of this paper were the small sample size and heterogeneity as this is still a novel surgical technique of the robotic-assisted technology. Another limitation was the short follow-up time, which might not reveal the re-revision rate of this technique. We hope that with future larger sample studies and the future development of a dedicated revision arthroplasty software system, this technique can be improved and widely used.

## Conclusion

Robot-assisted revision TKA is a promising technique to improve surgical outcomes by increasing the accuracy of implant placement, soft tissue protection and achieving a better-balanced knee. Further studies on this technology are needed to show that these benefits can be translated into an increased implant lifespan and patients' satisfaction. This paper described the methods of using robotic system in revision total knee arthroplasty from a variety of scenarios. However, future development of a revision arthroplasty software for the robotic arm may be needed.

## Data Availability

Not applicable.

## Abbreviations

TKA: Total Knee Arthroplasty
CT: Computer Tomographic
MARS: Metal Artefact Reduction Software
UKA: Unicompartmental Knee Arthroplasty
MPS: Mako™ Product Specialist
